# Awareness of Pelvic Floor Disorders Among Postpartum and Menopausal Women: A Cross-Sectional Study

**DOI:** 10.7759/cureus.92203

**Published:** 2025-09-13

**Authors:** Zaheera Saadia, Ghadah Alharbi, Latifah Y Almutlaq, Thekra A Alsamel, Summer A Alkhomairi, Reema M Alharbi, Ajyal Aljohani, Shaden M Alosaimi, Elaf S Alahmadi, Shahd Aliwaisi

**Affiliations:** 1 Gynecology, Qassim University, Buraidah, SAU; 2 Medicine, Qassim University, Buraidah, SAU; 3 Obstetrics and Gynecology, Qassim University, Buraidah, SAU; 4 Internal Medicine, Qassim University, Buraidah, SAU; 5 Medicine, Taibah University, Medina, SAU; 6 Medicine, Majmaah University, Riyadh, SAU; 7 Obstetrics and Gynecology, Maternity and Children Hospital, Madinah, SAU; 8 Medicine, Mansoura University, Mansoura, EGY

**Keywords:** pelvic floor disorder, pelvic floor dysfunction, pelvic organ prolapse, postpartum, saudi arabia, urinary incontinence, women’s health

## Abstract

Background

Pelvic floor dysfunction (PFD), including urinary incontinence (UI) and pelvic organ prolapse (POP), significantly affects the quality of life of women. Despite its prevalence, awareness remains suboptimal, particularly in understudied populations such as women in Saudi Arabia. This study assessed and compared the knowledge of PFD between postpartum and menopausal women in the central region.

Methods

A cross-sectional online survey was conducted among 374 women aged 18-70 years, classified as postpartum (women who had delivered a child within the past 12 months, regardless of breastfeeding status or menstrual cycle regularity) or menopausal (women aged 45 or above with at least 12 consecutive months of amenorrhea in the absence of other pathological or iatrogenic causes). The validated Prolapse and Incontinence Knowledge Questionnaire (PIKQ) was used, with scores categorized into high (POP ≥6, UI ≥10) and low (POP <6, UI <10) awareness. Descriptive statistics, independent samples t-tests, and chi-square tests were used for the analysis (IBM SPSS Statistics for Windows, Version 26 (Released 2019; IBM Corp., Armonk, New York), p<0.05).

Results

A total of 374 women participated, including 335 (89.6%) postpartum and 39 (10.4%) menopausal women. The mean age was 39.2 ± 11.7 years. High awareness of POP was observed in 263 (70.3%) participants, while 151 (40.4%) demonstrated high UI awareness. Postpartum women had significantly higher UI knowledge scores than menopausal women (mean = 8.57 vs. 7.51, p = 0.018); however, no significant difference was observed in POP knowledge (p = 0.566). Visiting a urologist or urogynecologist was more common in menopausal women (p = 0.001).

Conclusion

This study revealed a significant gap in UI awareness among menopausal women in the central region, despite the relatively high awareness of POP. Postpartum women demonstrated greater overall knowledge. These findings underscore the need for targeted educational initiatives across life stages to improve early recognition, reduce stigma, and encourage appropriate care of pelvic floor disorders.

## Introduction

Pelvic floor dysfunction (PFD) represents a significant health concern for postpartum and menopausal women due to anatomical and physiological changes associated with childbirth and the aging process, respectively. Recent studies underscore the multifactorial nature of PFD, emphasizing the roles of childbirth trauma, hormonal changes, and the overall physical health of women during these paramount life stages. In Saudi Arabia, where PFD remains underreported, a cross-sectional study found 60.2% of women exhibited PFD symptoms, with bowel (67.7%) and urinary dysfunction (44.1%) being particularly prevalent [1]. This underscores the critical need for improved awareness and healthcare strategies in the region.

Postpartum women face heightened vulnerability due to pelvic floor muscle damage during childbirth, with studies reporting up to 99.75% experiencing abnormal pelvic floor function shortly after delivery [2]. Conversely, menopausal women are predisposed to PFD due to estrogen deficiency, which weakens pelvic support structures [3].

Yet despite this physiological vulnerability, many women in both groups do not seek help. In a cohort study involving 64,650 middle-aged women, only 38% of those experiencing urinary incontinence (UI) discussed it with a healthcare provider, and just 13.9% received treatment [4]. Another study found that while 15% of individuals with stress urinary incontinence (SUI) sought medical assistance, just one-third of them were provided with guideline-recommended treatments [5]. This underreporting is often linked to embarrassment, stigma, or the misconception that PFD is a normal consequence of aging or childbirth [5].

Research indicates significant knowledge gaps across both groups, with many women lacking information on prevention, symptoms, and management. For instance, Neels et al. [6] found that 75% of peripartum and 68% of menopausal women expressed a need for better PFD education. In postpartum women, the vulnerability of the pelvic floor muscles and surrounding structures following childbirth is notably pronounced. A systematic review indicated that births, particularly vaginal deliveries, can lead to various forms of PFD, including UI and pelvic organ prolapse (POP), primarily caused by damage to the muscular and connective tissues of the pelvic floor during labor [7].

While some studies from major Saudi cities like Riyadh [1] (located in the central region) have begun to quantify the prevalence of PFD and assess knowledge levels, there remains a need for broader regional data across diverse populations. Understanding the awareness levels among women in different life stages, particularly postpartum and menopausal, is essential to inform regional health education and intervention strategies. Therefore, this study aims to assess and compare the level of awareness regarding PFD, focusing on UI and POP, between postpartum and menopausal women in the central region using the validated Prolapse and Incontinence Knowledge Questionnaire (PIKQ) [8]. By identifying knowledge gaps and associated factors, this research seeks to support the development of targeted educational programs that promote early recognition and management of pelvic floor disorders.

## Materials and methods

Study design

This study adopted a cross-sectional design aimed at comparing the level of knowledge about PFD between postpartum and menopausal women. The study utilized the PIKQ to assess participants' understanding of PFD, with a focus on UI and POP. Prior to implementation, permission was obtained from the original authors of the PIKQ questionnaire for both the English version [8] and the validated Arabic translation [9]. The full questionnaire is provided in the Appendices (Table 9).

Setting and participants

The study was conducted via an online survey distributed across social media platforms targeting women residing in the central region of Saudi Arabia. Participants were classified into two groups based on their physiological status: postpartum (women who had delivered a child within the past 12 months, regardless of breastfeeding status or menstrual cycle regularity) and menopausal (women aged 45 or above with at least 12 consecutive months of amenorrhea in the absence of other pathological or iatrogenic causes).

Inclusion and exclusion criteria

Eligible participants included women aged 18 to 70 years who could read and complete a questionnaire in Arabic or English. Women who refused participation, were below 18 years of age, or resided outside the central region were excluded from the study.

Data collection tool

The PIKQ, a validated instrument, was used to evaluate participants' knowledge. The tool comprises two 12-item subscales: one assessing knowledge of UI and the other focused on POP. Each correct response was scored as 1, while incorrect and "Don't Know" responses were scored as 0, resulting in total subscale scores ranging from 0 to 12. Its reliability and stability for measuring PFD-related knowledge have been empirically demonstrated, particularly in distinguishing proficiency levels among diverse populations [8]. For this study, the questionnaire was administered in both English and Arabic. To determine proficiency levels, we established cutoff scores for significant awareness at 80% (≥10 points) for the UI subscale and 50% (≥6 points) for the POP subscale, consistent with the standard scoring criteria outlined in the PIKQ validation study.

Sampling method and sampling size

A convenience sampling technique was employed. The questionnaire was disseminated through digital platforms commonly used by women in the target region, maximizing accessibility and reach. Participation was voluntary and anonymous. The sample size was determined using G*Power Software version 3.1.9.7 (Heinrich Heine University Düsseldorf, Düsseldorf, Germany), based on an independent samples t-test to compare the mean knowledge scores of UI and POP between postpartum and menopausal women. A medium effect size (Cohen's d = 0.5) was assumed based on prior literature indicating moderate differences in awareness levels between life stages [6,10]. With a power of 80% (1 - β = 0.80) and an alpha level of 0.05 (two-tailed), the minimum required sample size per group was 64 participants, totaling 128 participants. However, to account for potential non-response or incomplete surveys and to enhance statistical reliability, we aimed for a larger sample. Ultimately, 374 valid responses were collected, exceeding the minimum requirement and thereby improving the robustness of the analysis. The statistical power for the observed effect sizes was recalculated post hoc and found to exceed 90%, ensuring adequate sensitivity for detecting group differences.

Data management and statistical analysis

Data were entered into IBM SPSS Statistics for Windows, Version 26 (Released 2019; IBM Corp., Armonk, New York) for analysis. Descriptive statistics, including frequencies, means, and standard deviations, were calculated to summarize demographic characteristics and knowledge scores. Tests of normality indicated that the distribution of continuous variables deviated from normality; hence, nonparametric methods such as Spearman's correlation were used where appropriate. To compare knowledge levels between the postpartum and menopausal groups, independent samples t-tests were conducted for the UI and POP subscale scores. Significance was established at a p-value of <0.05. Responses were further explored through principal component analysis to examine the internal structure of the questionnaire scales.

Ethical considerations

This study received approval from the Qassim University Institutional Review Board (approval number: 607/46/7218). All participants provided informed consent by voluntarily completing the questionnaire. Confidentiality and anonymity of participant responses were strictly maintained throughout the study.

## Results

Demographic characteristics

A total of 374 women participated in this study, comprising 335 (89.6%) postpartum women and 39 (10.4%) menopausal women. The mean age of participants was 39.20 years (SD=11.66). Regarding educational attainment, the majority of participants (n=261, 69.8%) held a bachelor's degree, while 33 (8.8%) had a master's degree or higher. Most respondents (n=341, 91.2%) did not work in a medical field. Household income varied, with 162 (43.3%) participants reporting a monthly income of less than 6000 SAR, and only 12 (3.2%) earning more than 24,000 SAR (Table 1).

**Table 1:** Demographic and Baseline Characteristics of the Study Participants (N=374)

Characteristic	Frequency (n)	Percentage (%)
Physiological Status		
Postpartum	335	89.6
Menopausal	39	10.4
Work in the Medical Field		
Yes	33	8.8
No	341	91.2
Highest Education Level		
Elementary or Middle School	9	2.4
High School	54	14.4
Diploma	17	4.5
Bachelor's Degree	261	69.8
Master's Degree or Higher	33	8.8
Monthly Household Income		
<6000 SAR	162	43.3
6000–12000 SAR	129	34.5
12000–24000 SAR	71	19
>24000 SAR	12	3.2

As shown in Table 2, while the majority of participants correctly identified several aspects of PFD, notable misconceptions and knowledge gaps persisted across both the UI and POP subscales.

**Table 2:** Participant Responses to Individual Items From the Urinary Incontinence (UI) and Pelvic Organ Prolapse (POP) Knowledge Questionnaire (PIKQ) Correct answers are marked with (Correct).

Item Statement	Response	n	%
Urinary Incontinence (UI)			
UI1. UI is more common in young women than old women	Agree	61	16.30%
	Disagree (Correct)	249	66.60%
	Don't know	64	17.10%
UI2. Women are more likely than men to leak urine	Agree (Correct)	293	78.30%
	Disagree	30	8.00%
	Don't know	51	13.60%
UI3. Other than pads and diapers, little can be done to treat leakage	Agree	45	12.00%
	Disagree (Correct)	250	66.80%
	Don't know	79	21.10%
UI4. It is NOT important to diagnose the type of leakage before treatment	Agree	57	15.20%
	Disagree (Correct)	268	71.70%
	Don't know	49	13.10%
UI5. Many things can cause urine leakage	Agree (Correct)	339	90.60%
	Disagree	5	1.30%
	Don't know	30	8.00%
UI6. Certain exercises can help control UI	Agree (Correct)	333	89.00%
	Disagree	0	0.00%
	Don't know	41	11.00%
UI7. Some medications may cause UI	Agree (Correct)	171	45.70%
	Disagree	27	7.20%
	Don't know	176	47.10%
UI8. Once people start to leak urine, they can’t control it again	Agree	49	13.10%
	Disagree (Correct)	256	68.40%
	Don't know	69	18.40%
UI9. Doctors can do bladder testing to diagnose UI	Agree (Correct)	274	73.30%
	Disagree	6	1.60%
	Don't know	94	25.10%
UI10. Surgery is the only treatment for UI	Agree	39	10.40%
	Disagree (Correct)	206	55.10%
	Don't know	129	34.50%
UI11. Giving birth many times may lead to UI	Agree (Correct)	253	67.60%
	Disagree	32	8.60%
	Don't know	89	23.80%
UI12. Most people who leak urine can be cured/improved with treatment	Agree (Correct)	279	74.60%
	Disagree	16	4.30%
	Don't know	79	21.10%
Pelvic Organ Prolapse (POP)			
POP1. POP is more common in young women than old women	Agree	47	12.60%
	Disagree (Correct)	236	63.10%
	Don't know	91	24.30%
POP2. Giving birth many times may lead to POP	Agree (Correct)	277	74.10%
	Disagree	30	8.00%
	Don't know	67	17.90%
POP3. POP can happen at any age	Agree (Correct)	180	48.10%
	Disagree	51	13.60%
	Don't know	143	38.20%
POP4. Certain exercises can stop POP from worsening	Agree (Correct)	290	77.50%
	Disagree	22	5.90%
	Don't know	62	16.60%
POP5. POP symptoms may include pelvic heaviness/pressure	Agree (Correct)	271	72.50%
	Disagree	11	2.90%
	Don't know	92	24.60%
POP6. A doctor can diagnose POP through examination	Agree (Correct)	306	81.80%
	Disagree	7	1.90%
	Don't know	61	16.30%
POP7. Once a patient has POP, little can be done to help	Agree	56	15.00%
	Disagree (Correct)	189	50.50%
	Don't know	129	34.50%
POP8. Daily heavy lifting can lead to POP	Agree (Correct)	243	65.00%
	Disagree	30	8.00%
	Don't know	101	27.00%
POP9. Surgery is one type of treatment for POP	Agree (Correct)	245	65.50%
	Disagree	16	4.30%
	Don't know	113	30.20%
POP10. Blood tests can diagnose POP	Agree	54	14.40%
	Disagree (Correct)	107	28.60%
	Don't know	213	57.00%
POP11. A pessary (rubber ring) can treat POP symptoms	Agree (Correct)	109	29.10%
	Disagree	18	4.80%
	Don't know	247	66.00%
POP12. Obese people are less likely to get POP	Agree	47	12.60%
	Disagree (Correct)	152	40.60%
	Don't know	175	46.80%

Awareness of UI and POP

The mean score for UI knowledge was 8.46 (SD=2.64) out of 12, while the mean POP knowledge score was 6.96 (SD=3.11) out of 12. The overall PIKQ total score averaged 15.42 (SD=5.13) out of 24 (Table 3).

**Table 3:** Knowledge Scores for Pelvic Organ Prolapse (POP), Urinary Incontinence (UI), and Total PIKQ PIKQ: Prolapse and Incontinence Knowledge Questionnaire

Variable	Mean	Standard Deviation (SD)	Minimum	Maximum
POP Knowledge Score (out of 12)	6.96	3.11	0	12
UI Knowledge Score (out of 12)	8.46	2.64	0	12
Total PIKQ Score (out of 24)	15.42	5.13	0	24

Based on predetermined thresholds (≥10 points for UI and ≥6 points for POP), 40.4% (n=151) of participants demonstrated high awareness of UI, whereas 70.3% (n=263) showed high awareness of POP.

Comparison between postpartum and menopausal women

When comparing knowledge levels between the two groups, postpartum women demonstrated higher scores across all domains. The mean POP knowledge score for postpartum women was 6.99 ± 3.12, compared to 6.69 ± 3.04 for menopausal women. This difference was not statistically significant (t = 0.574, p = 0.566; U = 6119, p = 0.515). The mean UI knowledge score was significantly higher among postpartum women compared to menopausal women (8.57 ± 2.61 vs. 7.51 ± 2.80, respectively; t = 2.379, p = 0.018; U = 5049, p = 0.019). The total PIKQ score was also higher among postpartum women (15.56 ± 5.08) compared to menopausal women (14.21 ± 5.42), although this difference was not statistically significant (t = 1.57, p = 0.117; U = 5608, p = 0.147). Due to non-normal distributions (Shapiro-Wilk, all p < 0.05), Mann-Whitney U tests were performed to confirm t-test results, with consistent conclusions (Table 4).

**Table 4:** Comparison of Knowledge Scores Between Postpartum and Menopausal Women *Statistically significant at p < 0.05. POP: Pelvic Organ Prolapse; UI: Urinary Incontinence; PIKQ: Prolapse and Incontinence Knowledge Questionnaire

Variable	Postpartum (n=335) Mean ± SD	Menopausal (n=39) Mean ± SD	Test Statistic (t)	p-value (t-test)	Mann-Whitney U	Z-score	p-value (Mann-Whitney U)
POP Knowledge Score	6.99 ± 3.12	6.69 ± 3.04	0.574	0.566	6119	-0.651	0.515
UI Knowledge Score	8.57 ± 2.61	7.51 ± 2.80	2.379	0.018*	5049	-2.34	0.019*
Total PIKQ Score	15.56 ± 5.08	14.21 ± 5.42	1.57	0.117	5608	-1.45	0.147

Regarding healthcare-seeking behavior, menopausal women were significantly more likely to have consulted a urologist or urogynecologist compared to postpartum women (p=0.001) (Table 5).

**Table 5:** Healthcare-Seeking Behavior Among Study Participants *Statistically significant at p < 0.05. The Pearson chi-square test was used.

Variable	Postpartum (n=335) (%)	Menopausal (n=39) (%)	Test Statistic (χ²)	p-value
Ever seen a urologist/urogynecologist	186 (55.5%)	32 (82.1%)	10.112	0.001*
History of urine leakage	86 (25.7%)	14 (35.9%)	1.865	0.172
History of pelvic organ prolapse	32 (9.6%)	7 (17.9%)	2.637	0.104
Treated for urine leakage	24 (7.2%)	4 (10.3%)	0.482	0.487
Treated for pelvic organ prolapse	15 (4.5%)	3 (7.7%)	0.788	0.375

No significant differences were found between the groups regarding the history of urine leakage (p=0.172), history of POP (p=0.104), treatment for prolapse (p=0.375), or treatment for urine leakage (p=0.487). In terms of awareness levels, 235 (70.1%) postpartum women and 28 (71.8%) menopausal women had high POP awareness, a difference that was not statistically significant (p = 0.831). However, postpartum women had significantly higher UI awareness compared to menopausal women (p=0.048) (Table 6).

**Table 6:** Comparison of Awareness Levels for POP and UI *Statistically significant at p < 0.05. The Pearson chi-square test was used. POP: Pelvic Organ Prolapse; UI: Urinary Incontinence

Awareness Type	Postpartum (n=335) (%)	Menopausal (n=39) (%)	Test Statistic (χ²)	p-value
High POP Awareness	235 (70.1%)	28 (71.8%)	0.045	0.831
High UI Awareness	141 (42.1%)	10 (25.6%)	3.926	0.048*

Spearman's correlation analysis indicated no significant relationship between total PIKQ scores and participant age (r=0.006, p=0.904) or the number of children (r=0.023, p=0.659) (Table 7).

**Table 7:** Spearman's Correlation Matrix for Key Variables POP: Pelvic Organ Prolapse; UI: Urinary Incontinence; PIKQ: Prolapse and Incontinence Knowledge Questionnaire

Variables	Age	PIKQ Score	POP Score	UI Score
Age	1	0.006	0.064	-0.054
PIKQ Score	0.006	1	-	-
POP Score	0.064	-	1	-
UI Score	-0.054	-	-	1

Multivariate logistic regression analysis was conducted to determine predictors of POP awareness. Age, employment in the medical field, educational level, and physiological status (postpartum vs. menopausal) were not found to significantly predict POP awareness (Table 8). The overall fit of the model was acceptable, with a Hosmer-Lemeshow test p-value of 0.824, indicating good calibration.

**Table 8:** Logistic Regression Analysis for Predictors of POP Awareness *Statistically significant at p < 0.05. POP: Pelvic Organ Prolapse

Predictor Variable	B	SE	Wald	p-value	OR (Exp(B))
Age	0.005	0.014	0.145	0.703	1.005
Working in the Medical Field	0.603	0.456	1.742	0.187	1.828
Postpartum vs. Menopausal Status	0.052	0.444	0.014	0.907	1.053
Highest Education Level	-	-	2.529	0.639	-

## Discussion

This study assessed and compared the awareness levels of PFDs, specifically UI and POP, among postpartum and menopausal women in the central region of Saudi Arabia. While awareness of POP was relatively high (70.3%), only 151 (40.4%) participants demonstrated high awareness of UI, with postpartum women scoring significantly higher in UI knowledge than menopausal women (p=0.018). These findings align with previous research indicating limited awareness of PFDs among women. For instance, a study by Malaekah et al. reported that 60.2% of women in Riyadh experienced PFDs, yet awareness and understanding of these conditions remained low [1]. Similarly, a national survey highlighted that 23.4% of Saudi women experienced POP, emphasizing the need for increased education on PFDs [10].

It has been reported previously that UI awareness is often lower than POP awareness, particularly among menopausal women, who may attribute symptoms to aging rather than treatable conditions [11]. The higher UI knowledge among postpartum women could be attributed to recent exposure to healthcare during pregnancy and childbirth, where PFD education is more likely to be provided [12,13]. The lack of correlation between age and PFD knowledge (r = 0.006, p = 0.904) contrasts with studies in Spain and Saudi Arabia, where older age was associated with greater symptom experience but not necessarily better knowledge, suggesting that cultural stigma may deter open discussions about PFDs in older populations [12,14]. This discrepancy may reflect cultural barriers to discussing pelvic health among older women in conservative settings or the homogeneity of our sample, where age did not significantly vary in exposure to PFD education. The low healthcare-seeking behavior observed, with only 29.3% of Saudi women with UI seeking medical advice [12], reflects broader trends in low- and middle-income countries, where shame and lack of access to specialized care perpetuate underreporting. Menopausal women in our study were more likely to consult specialists (p=0.001), likely due to symptom severity, yet treatment rates remained low.

This study has some limitations that should be acknowledged. First, the cross-sectional design restricts our ability to infer causal relationships between awareness levels and demographic or clinical variables. Second, the use of a convenience sampling method and online distribution may have introduced selection bias, potentially overrepresenting women who are younger, more educated, or more technologically literate. As a result, the findings may not be generalizable to all postpartum and menopausal women across Saudi Arabia. Third, self-reported responses to sensitive topics such as UI and POP are subject to recall and social desirability bias, which may have led to underreporting or misclassification. Additionally, the study did not explore reproductive history variables such as parity, which may influence PFD awareness in other populations. Finally, the study did not explore cultural attitudes, stigma, or psychological barriers that could influence both awareness and willingness to seek care for pelvic floor disorders, factors that may be particularly relevant in conservative societies.

## Conclusions

This study highlights a significant disparity in the awareness of PFDs between postpartum and menopausal women in the central region, with postpartum women demonstrating notably higher knowledge of UI. While awareness of POP was relatively high across both groups, UI awareness remained suboptimal, particularly among menopausal women. These findings point to the urgent need for tailored educational interventions aimed at improving understanding of PFDs, especially among older women who may be less engaged with routine reproductive healthcare services. Integrating pelvic floor health education into maternal care, menopause counseling, and primary healthcare encounters could bridge existing knowledge gaps, reduce stigma, and encourage timely medical consultation. Future research should explore the impact of cultural perceptions, health literacy, and healthcare access on women's understanding and management of PFDs. Such efforts are essential to empower women across life stages with the knowledge and resources necessary to preserve pelvic health and improve overall well-being.

## Appendices

English and Arabic versions of the Prolapse and Incontinence Knowledge Questionnaire (PIKQ)

**Table 9:** Pelvic Floor Disorders Awareness Questionnaire Credits: Shah et al. [8] for the English version and Al-Kharabsheh et al. [9] for the Arabic version.

Section 1: Demographic Information		
English Text	Arabic Translation	
Age ______ years	العمر ______ سنة	
Which describes you?	ما الوصف الذي ينطبق عليك؟	
☐ Postpartum (regular menstruation)	☐ بعد الولادة (الحيض المنتظم)	
☐ Menopausal (≥45 years, no menstruation 12+ months)	☐ انقطاع الطمث (≥٤٥ سنة، بدون حيض لأكثر من ١٢ شهرًا)	
Do you work in the medical field?	هل تعمل في المجال الطبي؟	
☐ Yes	☐ نعم	
☐ No	☐ لا	
Monthly household income (SAR)	الدخل الشهري للأسرة (ريال سعودي)	
☐ <6,000	☐ أقل من ٦٬٠٠٠	
☐ 6,000–12,000	٦٬٠٠٠–١٢٬٠٠٠☐	
☐ 12,000–24,000	١٢٬٠٠٠–٢٤٬٠٠٠☐	
☐ >24,000	☐ أكثر من ٢٤٬٠٠٠	
Highest education level	أعلى مستوى تعليمي	
☐ Elementary/Middle School	☐ ابتدائي/متوسط	
☐ High School	☐ ثانوي	
☐ Diploma	☐ دبلوم	
☐ Bachelor’s Degree	☐ درجة البكالوريوس	
☐ Master’s/PhD	☐ ماجستير/دكتوراه	
Section 2: Healthcare History		
Have you ever seen a urologist/urogynecologist?	هل سبق أن زرت طبيب مسالك بولية/طبيب أمراض نسائية؟	
☐ Yes	☐ نعم	
☐ No	☐ لا	
Have you ever had urine leakage?	هل سبق أن عانيتِ من تسرب البول؟	
☐ Yes	☐ نعم	
☐ No	☐ لا	
Have you ever had pelvic organ prolapse?	هل سبق أن عانيتِ من تدلي أعضاء الحوض؟	
☐ Yes	☐ نعم	
☐ No	☐ لا	
Have you been treated for pelvic organ prolapse?	هل تلقيتِ علاجًا لتدلي أعضاء الحوض؟	
☐ Yes	☐ نعم	
☐ No	☐ لا	
Have you been treated for urine leakage?	هل تلقيتِ علاجًا لتسرب البول؟	
☐ Yes	☐ نعم	
☐ No	☐ لا	
Section 3: Knowledge Assessment		
Instructions: Answer 'True', 'False', or 'Don’t Know'.		
English Question	Arabic Translation	Response Options
Women are more likely than men to leak urine.	النساء أكثر عرضة لتسرب البول من الرجال.	☐ True ☐ False ☐ Don’t Know
Many things can cause urine leakage.	يمكن أن تسبب العديد من العوامل تسرب البول.	☐ True ☐ False ☐ Don’t Know
Certain exercises can help control urine leakage.	بعض التمارين يمكن أن تساعد في التحكم في تسرب البول.	☐ True ☐ False ☐ Don’t Know
Some medications may cause urinary leakage.	بعض الأدوية قد تسبب تسرب البول.	☐ True ☐ False ☐ Don’t Know
Doctors can perform bladder tests to diagnose urine leakage.	يمكن للأطباء إجراء اختبارات للمثانة لتشخيص تسرب البول.	☐ True ☐ False ☐ Don’t Know
Giving birth many times may lead to urine leakage.	الولادة المتكررة قد تؤدي إلى تسرب البول.	☐ True ☐ False ☐ Don’t Know
Most people who leak urine can be cured/improved with treatment.	معظم الأشخاص الذين يعانون من تسرب البول يمكن علاجهم أو تحسين حالتهم بالعلاج.	☐ True ☐ False ☐ Don’t Know
Urinary leakage is more common in young women.	تسرب البول أكثر شيوعًا بين النساء الشابات.	☐ True ☐ False ☐ Don’t Know
Other than pads/diapers, little can be done to treat leakage.	لا يمكن فعل الكثير لعلاج تسرب البول باستثناء استخدام الفوط الصحية أو الحفاضات.	☐ True ☐ False ☐ Don’t Know
It is NOT important to diagnose the type of leakage before treatment.	ليس من المهم تحديد نوع تسرب البول قبل العلاج.	☐ True ☐ False ☐ Don’t Know
Once leakage starts, it cannot be controlled again.	لا يمكن السيطرة على تسرب البول بمجرد أن يبدأ.	☐ True ☐ False ☐ Don’t Know
Surgery is the only treatment for urinary leakage.	الجراحة هي العلاج الوحيد لتسرب البول.	☐ True ☐ False ☐ Don’t Know
Giving birth many times may lead to pelvic organ prolapse.	الولادات المتكررة قد تؤدي إلى تدلي أعضاء الحوض.	☐ True ☐ False ☐ Don’t Know
Pelvic organ prolapse can happen at any age.	يمكن أن يحدث تدلي أعضاء الحوض في أي عمر.	☐ True ☐ False ☐ Don’t Know
Certain exercises can stop prolapse from worsening.	بعض التمارين يمكن أن تمنع تدهور التدلي.	☐ True ☐ False ☐ Don’t Know
Symptoms may include pelvic heaviness/pressure.	قد تشمل الأعراض ثقلًا أو ضغطًا في الحوض.	☐ True ☐ False ☐ Don’t Know
Doctors diagnose prolapse by pelvic examination.	يشخص الأطباء التدلي من خلال الفحص المهبلي.	☐ True ☐ False ☐ Don’t Know
Heavy daily lifting can lead to prolapse.	رفع الأوزان الثقيلة يوميًا قد يؤدي إلى تدلي أعضاء الحوض.	☐ True ☐ False ☐ Don’t Know
Surgery is one treatment for prolapse.	الجراحة هي أحد العلاجات للتدلي.	☐ True ☐ False ☐ Don’t Know
A pessary (rubber ring) can treat prolapse symptoms.	يمكن علاج أعراض التدلي باستخدام الحلقة المطاطية (الفرزجة).	☐ True ☐ False ☐ Don’t Know
Prolapse is more common after menopause.	التدلي أكثر شيوعًا بعد سن اليأس.	☐ True ☐ False ☐ Don’t Know
Doctors can diagnose prolapse with a blood test.	يمكن تشخيص تدلي أعضاء الحوض من خلال فحص دم.	☐ True ☐ False ☐ Don’t Know
Once prolapse occurs, little can be done to help.	بمجرد حدوث التدلي، لا يمكن فعل الكثير للمساعدة.	☐ True ☐ False ☐ Don’t Know
Obese people are less likely to get prolapse.	الأشخاص الذين يعانون من السمنة أقل عرضة للإصابة بالتدلي.	☐ True ☐ False ☐ Don’t Know
